# Exosomal double-stranded DNA as a biomarker for the diagnosis and preoperative assessment of pheochromocytoma and paraganglioma

**DOI:** 10.1186/s12943-018-0876-z

**Published:** 2018-08-23

**Authors:** Liang Wang, Ying Li, Xin Guan, Jingyuan Zhao, Liming Shen, Jing Liu

**Affiliations:** Stem Cell Clinical Research Center, National Joint Engineering Laboratory, Regenerative Medicine Center, The First Affiliated Hospital of Dalian Medical University, No. 193, Lianhe Road, Shahekou District, Dalian, Liaoning 116011 People’s Republic of China

**Keywords:** Double-stranded DNA, Exosomes, Diagnosis biomarker, Pheochromocytoma, Paraganglioma

## Abstract

Pheochromocytomas (PCCs) and paragangliomas (PGLs) are the most heritable endocrine tumors. Genetic testing for 12 driver susceptibility genes is recommended in all PCC and PGL cases. However, detection of somatic mutations in PCC and PGL remains unrealizable for genetic diagnosis and preoperative assessment. We compared the serum exosomal DNA and tumor tissue DNA from patients or mice with PCC or PGL and found double-stranded DNA (dsDNA) fragments in the circulating exosomes of patients with PCC or PGL. Exosomal dsDNA shared the same mutations in the susceptibility genes with that of the parent tumor cells. Moreover, our research showed that serum-derived exosomal dsDNA in PCC and PGL was highly consistent with the paired tumor genome. Our findings provide the first definitive evidence of the presence of exosomal dsDNA that can be used as a noninvasive genetic marker in one of the most effective somatic mutation screens for the diagnosis and preoperative assessment of PCCs and PGLs.

**Electronic supplementary material:**

The online version of this article (10.1186/s12943-018-0876-z) contains supplementary material, which is available to authorized users.

## Main text

Pheochromocytomas (PCCs) and paragangliomas (PGLs), the most heritable endocrine tumors, demonstrate major genetic driver events including germline and somatic mutations [1, 2]. Specific genotype–phenotype correlations have been established between susceptibility gene mutations and their clinical presentations [1, 3, 4]. Thus, assessment of these susceptibility genes for mutations is recommended for the early diagnosis and preoperative assessment of all PCCs and PGLs [5, 6].

Germline mutation testing for clinical diagnosis has become well established in recent decades [7]. Since more than one-third of PCC and PGL patients harbor only somatic mutations, monitoring of somatic mutations is recommended even for patients who are negative for germline mutations [1, 8]. However, it is difficult to detect somatic mutations prior to surgery, which limits genetic testing applications in practice.

Exosomes are an effective biomarker source independent of tissues and contain RNA, DNA, and proteins for noninvasive diagnosis [9]. However, the presence of DNA in exosomes is usually dependent on cell type, and the ability of the exosomal DNA to reflect mutational status of the parental tumor cells in PCC or PGL patients is largely unknown. Thus, in this study, we focused on exosomal DNA from PCC or PGL exosomes, and its potential as samples for screening somatic mutations in the parental tumor cells. This could serve as a promising noninvasive biomarker for clinical genetic diagnosis and preoperative assessment of PCC and PGL.

## Results and discussion

### PCC and PGL exosomes contain double-stranded genomic DNA

**Fig. 1:**
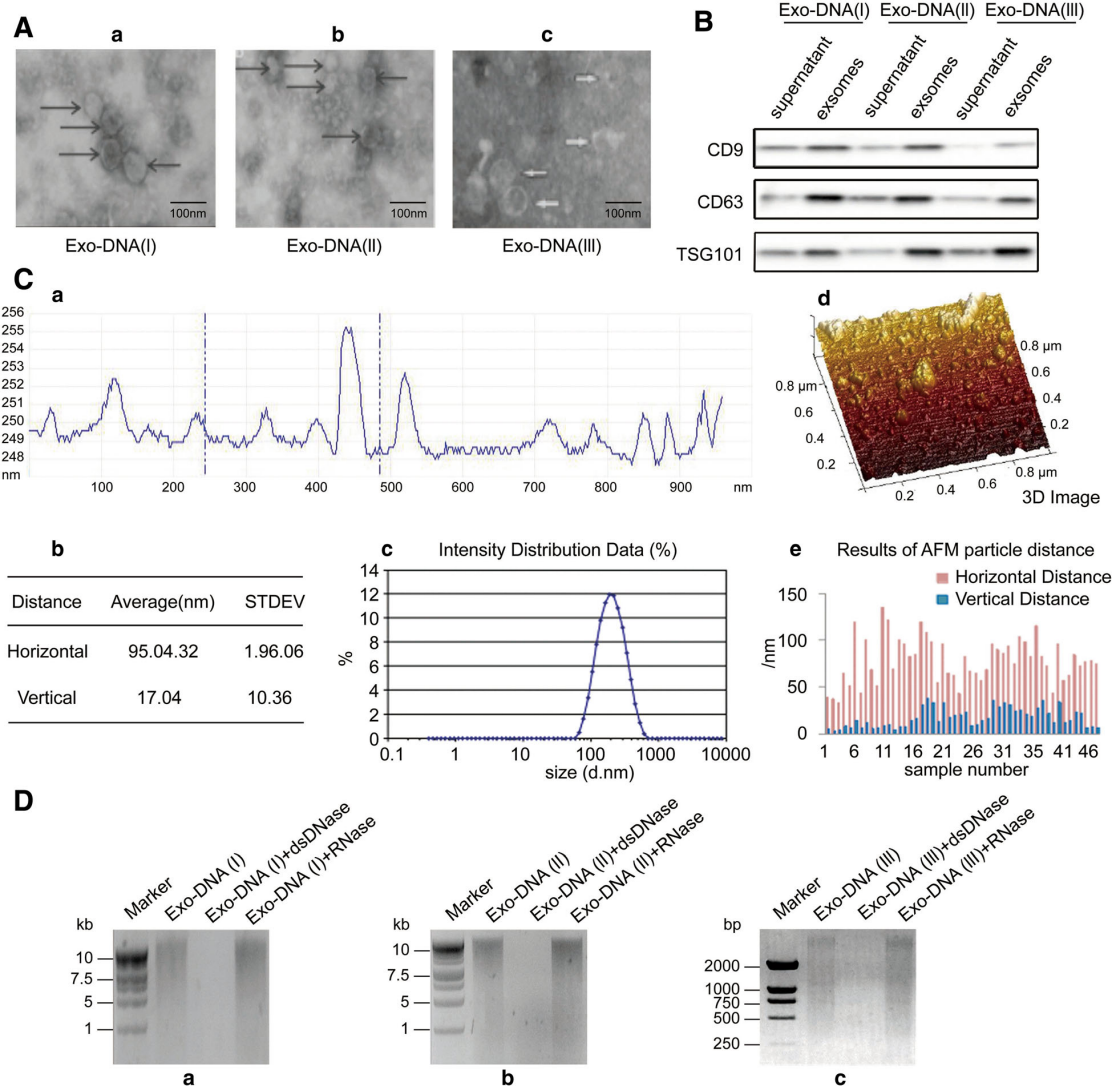
Exosomes contain double-stranded genomic DNA. **A** The characteristics of exosomes were determined by electron microscopy. **B** Exosomes were also characterized by the expression of exosome-specific CD9, CD63, and TSG101 using western blotting. **C** The presence and concentration of exosomes in the sera of PCC and PGL patients was detected using NanoDrop™ 2000 and Transmission electron microscopy (*n* = 15). **D** To exclude genomic DNA and RNA contamination after exosomal DNA extraction, the DNA eluted from PC12 cells (Exo-DNA I), mutated xenografts (Exo-DNA II), PCC or PGL tumors (Exo-DNA III), or the corresponding exosomes was treated with DNase I and RNase A

In order to evaluate the DNA carried by exosomes, we isolated exosomes from PC12 cell culture medium, the serum of mice implanted with mutated xenografts, and the serum of PCC or PGL patients. The characteristics of exosomes were assessed (Fig. 1A-C). For further characterization, we isolated the exosomal DNA from PC12 supernatants and digested it with dsDNase and RNase. We observed that the majority of DNA in PC12 exosomes (Exo-DNA (I)) was digested by dsDNase rather than RNase (Fig. 1D a). The same pattern was observed for exosomal DNA isolated from the serum of xenograft-implanted mice (Exo-DNA (II)) and the serum of PCC or PGL patients (Exo-DNA (III)) (Fig. 1D b and c). Taken together, these results suggest that dsDNA is the predominant form of DNA in exosomes of tumor cell supernatants and the sera of mice with xenografts and patients with PCC and PGL. Our novel finding is consistent with the presence of dsDNA in the exosomes of pancreatic cancer and prostate cancer cells, and may differ from those in astrocytes and glioblastoma cells because exosomal DNA packaging methods may differ among cancer types [9]. All the methods and materials used during this study are included in Additional file 1.

### PCC and PGL exosomes contain dsDNA with RET, VHL, HIF2A, and SDHB mutations

**Fig. 2:**
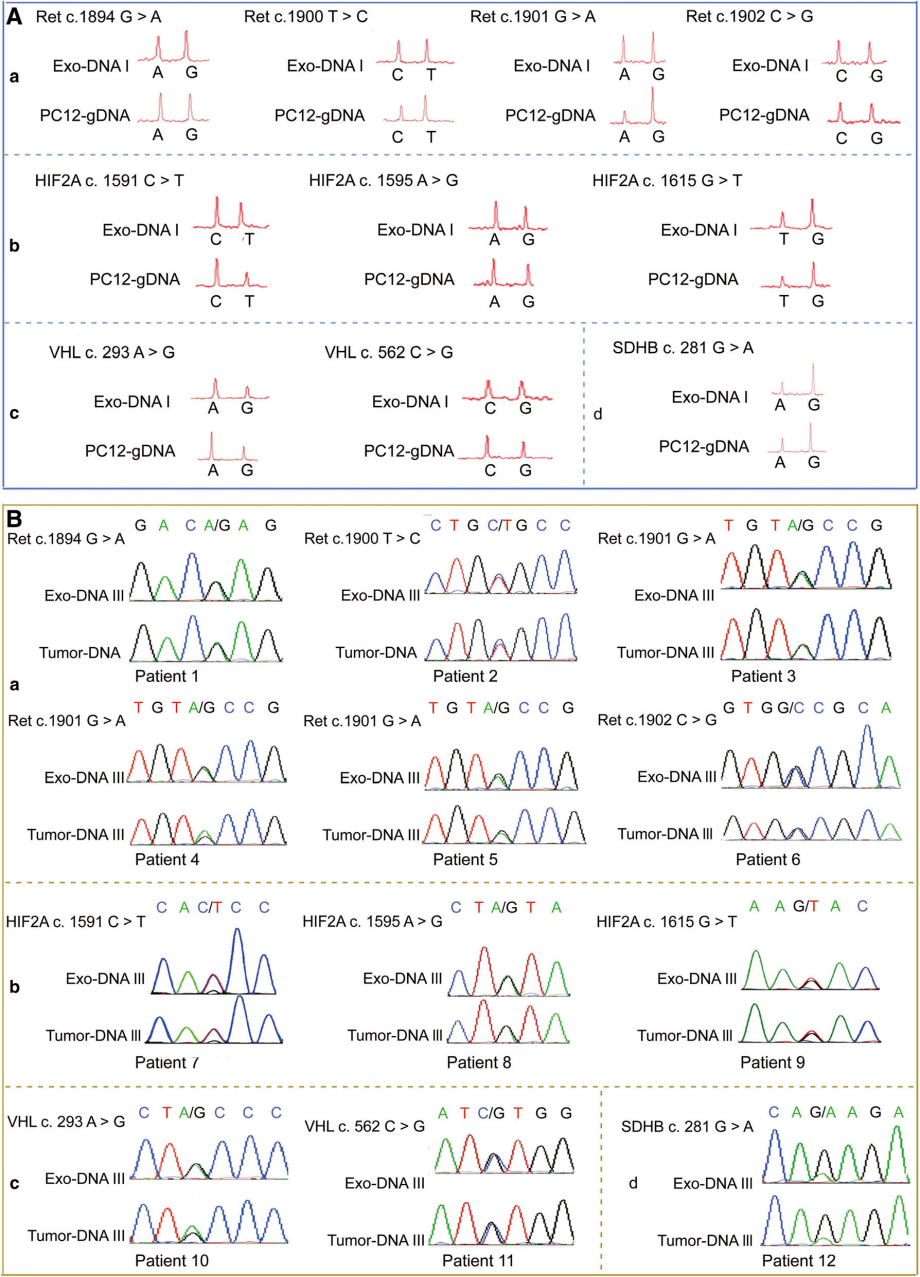
Exosomes contain mutated *RET, HIF2A, VHL,* and *SDHB* DNA. **A** iPLEX® mutation analysis of genomic DNA from PC12 and corresponding exosomes revealed the same heterozygous mutation pattern in *RET*, *HIF2A, VHL*, and *SDHB* mutations. **B** Sanger sequencing of serum exosome-derived DNA revealed *RET, VHL, HIF2A,* and *SDHB* mutations in tissues from PCC and PCL patients

*RET, HIF2A, VHL,* and *SDHB* are most frequently mutated genes in PCC and PGL and is usually monitoring for somatic mutations in sporadic PCC and PGL. To further explore somatic mutation testing in patients without germline mutations for PCC and PCL, we constructed plasmids expressing mutated human *RET* (c.1902C > G, c.1901G > A, c.1900 T > C, and c.1894G > A)*, HIF2A* (c.1615G > T, c.1595A > G, and c.1591C > T)*, VHL* (c.562C > G and c.293A > G)*,* and *SDHB* (c.281G > A*),* and used them to transfect PC12 cells. We found that the plasmid-encoded mutations were detected in both the transfected PC12 cells and in the exosomes from the supernatants (Fig. 2A a-d). To evaluate the feasibility of screening DNA from circulating exosomes for susceptibility gene mutations, we separately established stably transfected PC12 cell lines with the 10 mutations described above. We implanted the transfected PC12 cells subcutaneously in the flanks of nude mice, and harvested their sera when the tumors reached the maximum size after 30 days, to ensure isolation of sufficient circulating exosomes for analysis. We analyzed exosomal DNA from the serum for mutations by Sanger sequencing (Additional file 2: Table S1).

Based on our observations in tumor cells and the animal model, we hypothesize that human serum exosomes may contain information regarding the mutation of *RET, VHL, HIF2A,* and *SDHB* in their parental cells. We analyzed samples from 12 PCC or PGL patients whose somatic tumor mutations had been identified by genetic diagnosis (Additional file 3: Table S2). We found that *RET* (Fig. 2B *a*)*, HIF2A* (Fig. 2B *b*)*, VHL* (Fig. 2B *c*)*,* and *SDHB* mutations (Fig. 2B *d*) in serum exosomal DNA were definitively consistent with the somatic tumor mutations in patients with PCC or PGL. Collectively, these results provide evidence that serum exosomal dsDNA may serve as a primary somatic mutation diagnostic biomarker for PCC or PGL preoperative assessment.

### Serum Exosomes from PCC and PGL patients contain genomic dsDNA that covers all chromosomes

**Fig. 3:**
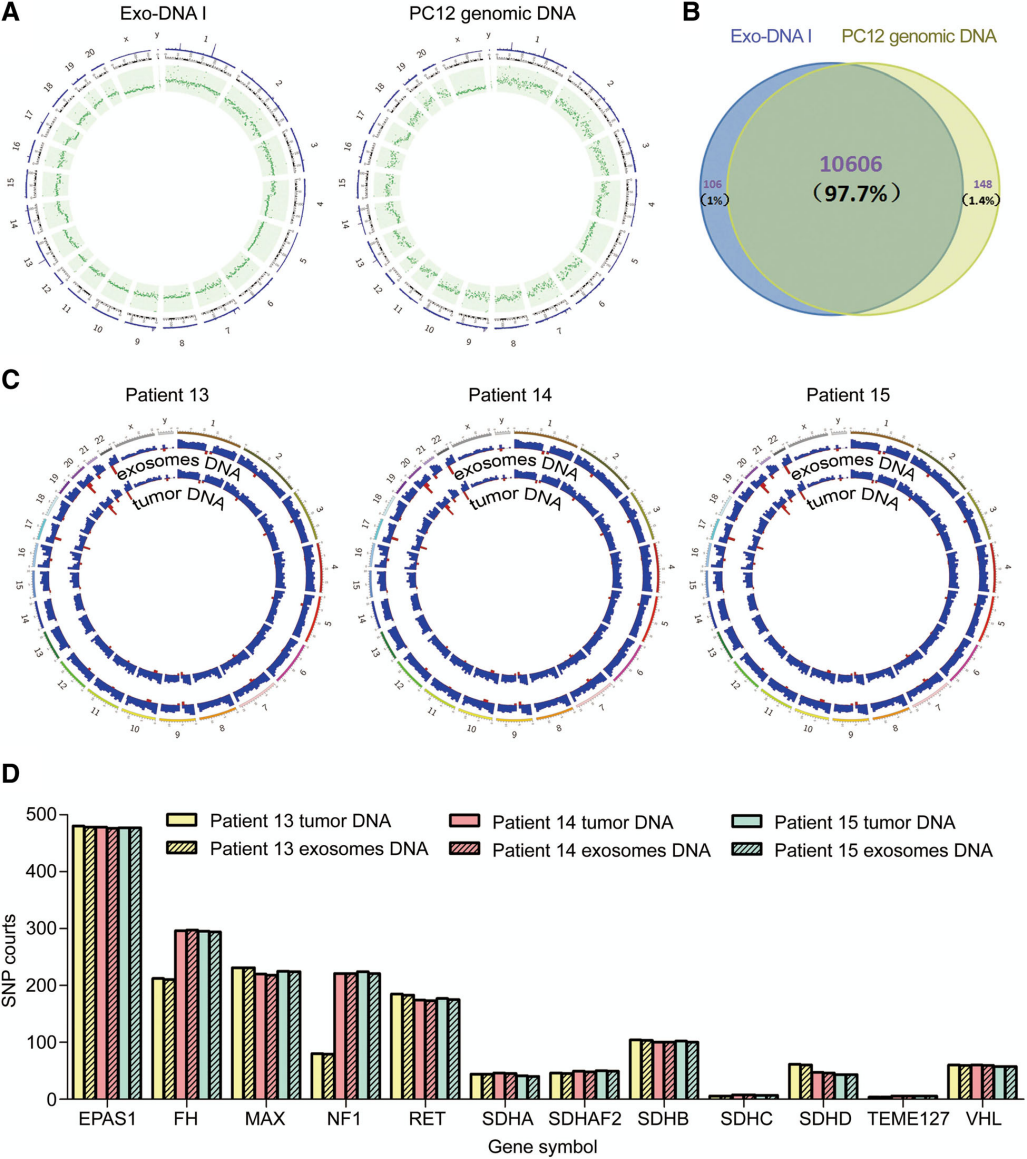
Serum-derived exosomes contain genomic DNA spanning all chromosomes. **a** The mutations in Exo-DNA I are displayed on a chromosome map generated using Circos (v0.67). The blue bar on the outer circle indicates the average depth of the sequence and the green dots in the inner ring represent the number of mutations. **b** Venn diagram of all SNPs shared by Exo-DNA I and PC12 genomic DNA. **c** Whole-genome sequencing was conducted on serum-derived exosomal DNA and the corresponding primary tumors from 3 patients. The inner and outer tracks indicate the SNP counts for exosomes and paired PCC or PGL samples, respectively. All chromosomes are represented in 10-Mbp-wide windows. The SNP counts in each region represent the total SNP count (0–20,000) for all loci in this region. **d** All common SNP counts of the 12 susceptibility genes for PCC and PGL are shown with their relative proportions

To further determine if the dsDNA from circulating exosomes reflects the mutational status of their parental tumor cells, we compared the exosomal DNA from PC12 supernatants with its genomic DNA. The results of whole-genome sequencing demonstrated that exosomal DNA covered 97.7% of the single nucleotide polymorphisms (SNPs) of the parent PC12 cells (Fig. 3a and b and Additional file 4: Table S3). In order to confirm these results, we compared the exosomal DNA and paired tumor tissues from 3 PCC or PGL patients. Importantly, our results revealed that the entire genome is covered by the exosomal DNA in an unbiased manner (Fig. 3c). We examined the 12 driver susceptibility gene mutations in exosomal DNA, and found that the concordance rates of mutations in the exosomal and tumor tissue DNA were as high as 97.6–100% (Fig. 3d and Additional file 5: Table S4-S7). Taken together, our results demonstrate a high degree of consistency between serum exosomal DNA and paired tumor genomic DNA in patients with PCC or PGL.

Thus, our analysis revealed that the bulk of serum-derived exosomes of patients with PCC or PGL contained dsDNA that spanned all chromosomes and resembled nuclear genomic DNA (Fig. 3c). The presence of dsDNA in exosomes allows detection of somatic mutations in susceptibility genes before surgery in patients with PCC or PGL.

## Conclusions

This is the first study to reveal that PCC and PGL exosomes contain dsDNA that can reflect the mutation status of susceptibility genes and cover nearly all chromosomes. The definitive evidence of exosomal dsDNA presence suggests its use as a noninvasive genetic marker in one of the most effective somatic mutation screens for the genetic diagnosis and preoperative assessment of PCCs and PGLs.

## Additional files

Additional file 1: Methods and materials used during this study. (DOCX 45 kb)

Additional file 2: **Table S1.** Mutations in nude mice. (DOCX 82 kb)

Additional file 3: **Table S2.** Clinical and genetic characteristic of the PCCs/PGLs patients. (DOCX 21 kb)

Additional file 4: **Table S3.** Common SNP in exo-DNA I and genomic DNA of PC12 cells. (XLS 1157 kb)

Additional file 5: **Table S4.** Common SNP of susceptibility genes in exo-DNA III and the parental tumor cells from patient 13. **Table S5.** Common SNP of susceptibility genes in exo-DNA III and the parental tumor celsl from patient 14. **Table S6.** Common SNP of susceptibility genes in exo-DNA III and the parental tumor cells from patient 15. **Table S7.** Common SNP counts in three of the patients. (ZIP 184 kb)

## Abbreviations

CTC: Circulating tumor cell
ctDNA: Circulating tumor DNA
dsDNA: Double-stranded DNA
NGS: Next-generation sequencing
PCC: Pheochromocytomas
PGL: Paragangliomas
SNP: Single nucleotide polymorphism
