# Supplementary material for: Exosomal double-stranded DNA as a biomarker for the diagnosis and preoperative assessment of pheochromocytoma and paraganglioma
Source: Mol Cancer. 2018 Aug 23;17:128. doi: 10.1186/s12943-018-0876-z (PMC6108141; doi:10.1186/s12943-018-0876-z)
Supplement: Supplementary file 1 — Methods and materials used during this study. (DOCX 45 kb) [file 12943_2018_876_MOESM1_ESM.docx]

**Methods**

**Patients and tissue collection**

This study included tumors from 15 patients with apparently sporadic PCC or PGL, who underwent surgery following genetic testing. Three of the tumors did not have germline and somatic mutations in the 12 known driver susceptibility genes; the other 12 tumors had *RET, VHL, HIF2A*, and *SDHB* somatic mutations (Table 1). The clinical characteristics and details of the PCC or PGL patients have been previously described [38]. Peripheral blood (10 ml) was collected from the patients into separator tubes immediately before surgery. The blood samples were subjected to centrifugation at 3,000 ×*g* for 10 min to isolate serum and prepare exosomal DNA. All research was conducted in accordance with the ethical guidelines of The First Affiliated Hospital of Dalian Medical University.

**Cell culture**

In order to avoid contamination of the exosomes with those present in fetal bovine serum (FBS) and horse serum (HS), we subjected the FBS and HS to ultracentrifugation overnight at 100,000 ×*g* to remove exosomes before using the sera to culture PC12 cells. All cell media included exosome-depleted serum. We cultured PC12 cells (ATCC® CRL-1721, American Type Culture Collection) in RPMI 1640 (Gibco) with 10% HS, 5% FBS, and 1% penicillin–streptomycin, with medium changes every 48 h. We passaged cells every 96 h when the cells were 70%–80% confluent and collected the conditioned medium (250 ml), which we stored at −80°C.

**PC12 cell transfection and infection**

We cultured PC12 cells up to 8 passages to 70% confluence in 10-cm dishes. Two hours after changing the medium for penicillin- and streptomycin-free medium with serum, we added transfection reagent mixed with plasmid in Opti-MEM (Thermo Fisher Scientific). After washing with phosphate-buffered saline (PBS), we changed the complete medium 48 h after transfection, which we collected (150 ml) and stored at −80°C. For the adenovirus infection, we counted and passaged PC12 cells into a 96-well plate. After 24 h, we used unloaded adenovirus (Origene) to conduct the preliminary experiment. Seventy-two hours later, we used fluorescence microscopy to validate that the infection efficiency was >90%. Next, we transfected with plasmid (10 μl) with *RET, VHL, HIF2A,* or *SDHB* mutations (Beijing BioLink Biology Technology). We used 30 mol adenovirus and reagent to infect PC12 cells. We screened for stable infection by fluorescence microscopy and collected medium (250 ml) for exosome preparation (stored at −80°C).

**Xenograft experiments**

We used 30 BALB/c nude mice (4–6 weeks old) for the animal xenograft experiments. We subcutaneously injected in the back flank skin of each mouse PC12 cells (7×10^7^ in 0.2 ml PBS) that were stably infected with plasmids expressing *RET, VHL, HIF2A,* or *SDHB* mutations. After 35 and paired venous blood were taken out to isolate genomic DNA for mutation analysis.

**Isolation of exosomes from cultured cells and serum**

Exosomes were isolated by sequential centrifugation from the conditioned medium collected from PC12 cells, the serum of nude mice implanted with mutated xenografts, or PCC or PGL patient serum. PC12 cells were grown to 70%–80% confluency. The medium was replaced with medium containing 10% FBS and 5% HS that had been depleted of exosomes by differential centrifugation (2.5 h at 100,000 ×*g*). Then, the conditioned medium was subjected to centrifugation at 3,000 rpm for 10 min at 4°C to remove cellular debris and dead cells. The supernatants were subjected to centrifugation at 100,000 ×*g* for 2.5 h at 4°C. The exosome pellets were washed with 1× PBS and subjected to centrifugation at 100,000 ×*g* for 70 min. The final exosome pellets were resuspended in 1× PBS. To isolate exosomes from the peripheral or portal blood, samples were subjected to centrifugation at 3,000 rpm for 15 min to separate the serum from the red blood cells. The collected plasma was centrifuged again at 3,000 rpm for 10 min, then isolated by high-speed centrifugation, as described above. The resulting exosome pellets were washed and resuspended as described. The exosomal protein contents with quantified by BAC (Tiangen, Beijing).

**Transmission electron microscopy analysis**

Exosomes (5 μl) were fixed with 4% paraformaldehyde (5 μl) at room temperature for 30 min and stored at −4°C overnight. We mixed the samples with ultrasound equipment before adding them to copper wire mesh where they settled for 30 min, then were negative-stained with phosphotungstic acid for 5 min. We observed the samples with a transmission electron microscope and captured images at 60,000× and 150,000× amplification.

**Western blotting analysis**

Exosomes were isolated as described above from PC12 cultures and the serum from PCC or PGL patients and nude mice with mutated xenografts. After isolation, we extracted and quantified the total protein from the exosomes (30 μl). We subjected total protein (30 μg) to western blotting according to standard protocols, then probed with anti-CD9 (ab92726, Abcam), anti-CD63 (ab108950, Abcam), and anti-TSG (ab30871, Abcam) to detect exosomes in the fractions.

**Extraction and identification of exosomal DNA**

Before extracting DNA from exosomes, according to the instructions of the DNeasy Blood & Tissue Kit (51304, Qiagen), we treated the samples with DNase I (10 μl; 73504, Qiagen) and RNase A (4 μl) at room temperature for 15 min to digest the external DNA and RNA, respectively. Then, we used 140 mmol/L EDTA to chelate the samples and treated with proteinase K (10 μl) at 56°C for 10 min to digest the DNase I, RNase A, and other external proteins. We quantified the extracted DNA with a NanoDrop™ 2000 and assessed its quality by agarose gel electrophoresis (1% agar, U = 100 v, I = 50 mA).

We incubated the samples with dsDNase (1 μl; EN0771, Thermo Fisher Scientific) to digest exosomal DNA (0.5 μg) and DNA from PC12 cells at 37°C for 2 min, then subjected the samples to agarose gel electrophoresis (1% agar, U = 100 v, I = 50 mA) for qualitative and relatively quantitative analysis of the total DNA. Samples that were not subjected to digestion were used as controls.

**iPLEX® mutation analysis**

Genomic DNA was isolated and purified using a genomic DNA separation kit (Qiagen). Quantitative DNA levels were determined as previously described using the Sequenom MassARRAY iPLEX® system (Agena), a mass spectrometry-based method. The mutations were calculated to give the detection samples. Specific primers were designed using Sequenom Online Tools (Assay Design Suite, AgenaCx) as previously described.

**NGS**

We performed whole-genome sequencing of the DNA (30 μl) extracted from 3 PCC or PGL patient tumors and their serum exosomes. The genomic DNA from PC12 cells and their exosomes were also assayed by NGS according to a standard operating procedure.

**Dynamic light scattering (DLS) analysis of exosomes**

We eliminated exosomal protein with proteinase K according to the manufacturer's instructions (DNA isolation kit, Qiagen). We measured the sizes of the exosomes with a Zetasizer (Malvern Panalytical). Exosomes (diluted 1:200 in PBS) were analyzed with an equilibration time of 70 s at a constant temperature of 25°C. A laser beam (549 nm) was applied to the exosomes suspension and the scattered light was detected by an avalanche photodiode detector at 173° with noninvasive back scattering optics. The average of the 3 measurements was used to determine the sizes of the exosomes.

**Bioinformatics alignment analysis**

We illustrated the sequencing depth and distribution of mutations with Circos software (v0.67). The alignment result file (BAM) was used to calculate the depth of the whole-genome sequencing at each locus. We simultaneously applied the mutation analysis results file (VCF) to calculate the mutation positions in the whole genome.
