# Supplementary material for: Exosomal double-stranded DNA as a biomarker for the diagnosis and preoperative assessment of pheochromocytoma and paraganglioma
Source: Mol Cancer. 2018 Aug 23;17:128. doi: 10.1186/s12943-018-0876-z (PMC6108141; doi:10.1186/s12943-018-0876-z)
Supplement: Supplementary file 2 — Table S1. Mutations in nude mice. (DOCX 82 kb) [file 12943_2018_876_MOESM2_ESM.docx]

| **Table S1 Mutations in nude mice** | | | | |  |  |
| --- | --- | --- | --- | --- | --- | --- |
|  | Xenograft Group  I male | Exo-DNA II Group  I male | Sensitivity | Xenograft Group II female | Exo-DNA II Group II female | Sensitivity |
| RET (c.1902C>G) | C/G (2/2) | C/G (2/2) | 95% | C/G (2/2) | C/G (2/2) | 100% |
| RET (c.1901G>A) | G/A (2/2) | G/A (2/2) |  | G/A (2/2) | G/A (2/2) |  |
| RET (c.1900T>C) | T/C (2/2) | T/C (2/2) |  | T/C (2/2) | T/C (2/2) |  |
| RET (c.1894G>A) | G/A (2/2) | G/A (2/2) |  | G/A (2/2) | G/A (2/2) |  |
| HIF2A (c.1615G>T) | G/T (2/2) | G/T (2/2) |  | G/T (2/2) | G/T (2/2) |  |
| HIF2A (c.1595A>G) | A/G (2/2) | A/G (1/2) |  | A/G (2/2) | A/G (2/2) |  |
| HIF2A (c.1591C>T) | C/T (2/2) | C/T (2/2) |  | C/T (2/2) | C/T (2/2) |  |
| VHL (c.562C>G) | C/G (2/2) | C/G (2/2) |  | C/G (2/2) | C/G (2/2) |  |
| VHL (c.293A>G) | A/G (1/2) | A/G (1/2) |  | A/G (1/2) | A/G (1/2) |  |
| SDHB (c.281G>A) | G/A (2/2) | G/A (2/2) |  | G/A (2/2) | G/A (2/2) |  |

Sanger sequencing of genomic DNA from the tumors and serum exosomes of mice implanted with mutated xenografts confirmed that they shared the same mutation status for *RET, HIF2A, VHL,* and *SDHB.* In total, 40 mice were divided into 10 groups according to their mutations. Group I represents males (n = 2/group) and Group II represents females (n = 2/group).
