# Supplementary material for: Exosomal double-stranded DNA as a biomarker for the diagnosis and preoperative assessment of pheochromocytoma and paraganglioma
Source: Mol Cancer. 2018 Aug 23;17:128. doi: 10.1186/s12943-018-0876-z (PMC6108141; doi:10.1186/s12943-018-0876-z)
Supplement: Supplementary file 3 — Table S2. Clinical and genetic characteristic of the PCCs/PGLs patients. (DOCX 21 kb) [file 12943_2018_876_MOESM3_ESM.docx]

| **Table S2 Clinical and genetic characteristic of the PCCs/PGLs patients** | | | | | | | | | |
| --- | --- | --- | --- | --- | --- | --- | --- | --- | --- |
| **Sample ID** | **Sex** | **Age at diagnosis** | **Benign/ Malignant** | **Familial/Sporadic** | **Tumor size(cm)** | **Tumor location** | **Tumor type** | **Somatic Mutations** | **Germline Mutations** |
| 1 | M | 42 | B | S | 2.5 | R | PCCs | RET c.1894G>A p. G632K | Negative |
| 2 | F | 51 | B | S | 6.8 | R | PCC | RET c.1900T>C p.C634R | Negative |
| 3 | M | 36 | B | S | 5.8 | L | PCCs | RET c.1901G>A p.C634Y | Negative |
| 4 | M | 73 | B | S | 3 | L | PCCs | RET c.1901G>A p.C634Y | Negative |
| 5 | F | 59 | B | S | 4 | R | PCCs | RET c.1901G>A p.C634Y | Negative |
| 6 | F | 64 | B | S | 1.5 | L | PCCs | RET c.1902C>G p.C634W | Negative |
| 7 | F | 31 | B | S | 5.1 | L | PGLs | HIF2A c.1591C>T p. P531S | Negative |
| 8 | F | 53 | B | S | 4.2 | R | PCCs | HIF2A c.1595A>G p. Y532C | Negative |
| 9 | M | 46 | B | S | 4.6 | L | PCCs | HIF2A c.1615G>T p. D539Y | Negative |
| 10 | M | 41 | B | S | 2.7 | L | PCCs | VHL c.293A>G p. Y98C | Negative |
| 11 | M | 23 | B | S | 3.8 | R | PCCs | VHL c.562C>G p. L188V | Negative |
| 12 | M | 40 | Ma | S | 3.2 | R | PCCs | SDHB c.281G>A p. R94K | Negative |
| 13 | M | 46 | B | S | 4.2 | R | PCCs | Negative | Negative |
| 14 | M | 73 | B | S | 3.5 | R | PGLs | Negative | Negative |
| 15 | M | 22 | B | S | 4.8 | R | PCCs | Negative | Negative |
| M: male, F: female, B: benign, PCCs: pheochromocytoma, PGLs: paraganglioma (extra pheochromocytoma), R: right, L: left, B: benign, Ma: malignant, S: sporadic | | | | | | | | | |
